# Clinical Infections, Antibiotic Resistance, and Pathogenesis of *Staphylococcus haemolyticus*

**DOI:** 10.3390/microorganisms10061130

**Published:** 2022-05-31

**Authors:** Hala O. Eltwisy, Howida Omar Twisy, Mahmoud HR Hafez, Ibrahim M. Sayed, Mohamed A. El-Mokhtar

**Affiliations:** 1 Department of Microbiology, Faculty of Science, Beni-Suef University, Beni-Suef 62521, Egypt; hala.omar@aun.edu.eg; 2 Department of Dermatology, Venereology and Andrology, Assiut University, Assiut 71515, Egypt; d.howida@aun.edu.eg; 3 International Scholar, African Leadership Academy, 1050 Printech Road, Laser Park, Honeydew, Johannesburg 2040, South Africa; mhafez21@alastudents.org; 4 Department of Medical Microbiology and Immunology, Faculty of Medicine, Assiut University, Assiut 71515, Egypt; 5 Department of Pathology, School of Medicine, University of California, San Diego, CA 92093, USA; 6 Microbiology and Immunology Department, Faculty of Pharmacy, Sphinx University, Assiut 71515, Egypt

**Keywords:** *S. haemolyticus*, pathogenesis, antibiotic resistance, biofilm, virulence factors, clinical infections

## Abstract

*Staphylococcus haemolyticus (S. haemolyticus*) constitutes the main part of the human skin microbiota. It is widespread in hospitals and among medical staff, resulting in being an emerging microbe causing nosocomial infections. *S. haemolyticus*, especially strains that cause nosocomial infections, are more resistant to antibiotics than other coagulase-negative Staphylococci. There is clear evidence that the resistance genes can be acquired by other Staphylococcus species through *S. haemolyticus*. Severe infections are recorded with *S. haemolyticus* such as meningitis, endocarditis, prosthetic joint infections, bacteremia, septicemia, peritonitis, and otitis, especially in immunocompromised patients. In addition, *S. haemolyticus* species were detected in dogs, breed kennels, and food animals. The main feature of pathogenic *S. haemolyticus* isolates is the formation of a biofilm which is involved in catheter-associated infections and other nosocomial infections. Besides the biofilm formation, *S. haemolyticus* secretes other factors for bacterial adherence and invasion such as enterotoxins, hemolysins, and fibronectin-binding proteins. In this review, we give updates on the clinical infections associated with *S. haemolyticus*, highlighting the antibiotic resistance patterns of these isolates, and the virulence factors associated with the disease development.

## 1. Introduction

Coagulase-negative Staphylococci (CoNS) constitute the main microbiota of the skin. These pathogens were underestimated and a distinct species identification was not included in many microbiology laboratories [1]. Only the coagulase-positive *S. aureus* was considered pathogenic and therefore gained great interest and thoroughly analyzed in different studies. In the late 1960s, one of the CoNS, *S. saprophyticus*, was observed in patients with urinary tract infections (UTIs) [2]. Later, the first CoNS infections were identified in the 1970s in patients with invasive and indwelling medical devices [3,4]. The advances in diagnostic protocols and molecular techniques enabled more accurate identification of the other species in the genus *Staphylococci* [5]. Scientists have observed increasing numbers of CoNS infections. In the USA between 1980 and 1989, CoNS causing nosocomial bacteremia increased from 9 to 27% [6]. *Staphylococci* species are phylogenetically a very coherent group. The average nucleotide identity values of *S. aureus* versus CoNS such as *S. epidermidis* and *S. haemolyticus* is approximately 75%, showing their close genetic association [7,8].

*S. haemolyticus* is a part of skin microflora and one of the main species of CoNS [9]. This species accounts for 10–20% of clinical CoNS infections [10] and is the second-highest species of CoNS in frequency and importance among isolates from clinical infections [11]. There are several clinical infections recorded with *S. haemolyticus* including bacteremia, meningitis, eye infections, skin infections, peritonitis, urinary tract infections, and male genital dysfunction [12,13]. Furthermore, *S. haemolyticus* strains were isolated from dogs and dogs’ owners suggesting a possibility of zoonotic transmission [14]. A characteristic feature of *S. haemolyticus* is the formation of biofilms, which are crucial for the development of infections [15]. Furthermore, *S. haemolyticus* produces several toxins and invasive enzymes that help in bacterial pathogenesis by changing the host immune responses and inducing damage in the host cells [15].

*S. haemolyticus* is an emerging pathogen causing nosocomial infections. The factors which cause the survival and spread of *S. haemolyticus* in hospitals are not well defined [16]. The genome of *S. haemolyticus* causing hospital infections is characterized by the abundance of insertion sequences, and resistance to several antibiotics [17,18].

In the absence of appropriate diagnosis and management of infections caused by *S. haemolyticus*, resistant strains of this pathogen can spread to other hospital settings, and probably to the community [17].

In this review, we give updates on the clinical infections associated with *S. haemolyticus,* antibiotic resistance patterns of these isolates, and the virulence factors associated with the disease development.

## 2. Clinical Infections Associated with *S. haemolyticus*

**Figure 1:**
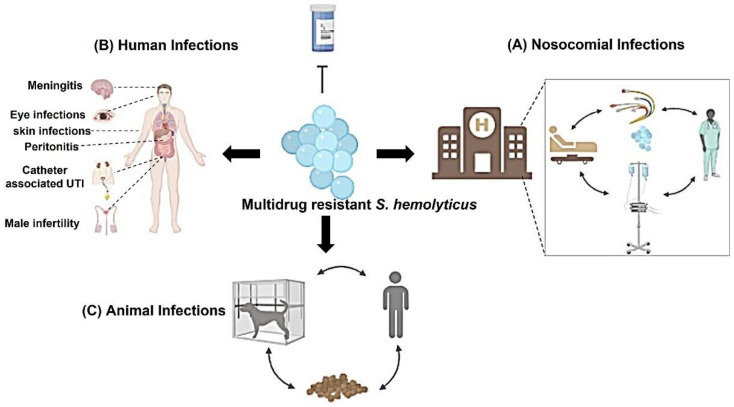
Infections associated with *S. haemolyticus*: Several infections are associated with *S. haemolyticus* isolates. *S. haemolyticus* causes nosocomial infections that can be spread among health care personnel, medical devices, catheters, and patients. In addition, several clinical human infections are recorded with *S. haemolyticus* such as eye infections, bacteremia, UTIs, male infertility, etc. Human infections are considered nosocomial infections if the infections are acquired in the hospitals. Moreover, *S*. *haemolyticus* infect animals such as dogs and infection can spread throughout the animal, owner, kennel breed, and animal food.

There are several clinical manifestations recorded with *S. haemolyticus* infections such as bloodstream infections, ocular infections, epididymo-orchitis, chronic prostatitis, UTI, etc. Furthermore, it is an important pathogen associated with hospital-acquired infections (Figure 1). Other complications were also recorded especially in immunocompromised patients such as septicemia, peritonitis, and otitis [12,13].

### 2.1. Bloodstream Infections (BSIs)

*S.aureus* and several species of CoNS such as *S. hominis*, *S. haemolyticus* and *S. epidermidis* cause BSIs in cancer patients [18]. *S. haemolyticus* causes bacteremia following the central catheter-related bloodborne infection [19,20], and it causes septicemia among neutropenic patients in intensive care units (ICU) [21] and renal dialysis catheter-related sepsis (CRS) [22]. *S. haemolyticus* causing BSIs are highly resistant to antibiotics, some isolates such as methicillin-resistant *S. haemolyticus* (MRSH) can cause severe complications and death [23,24]. The antimicrobial agents effective against multidrug-resistant *S. haemolyticus* are limited. Vancomycin and daptomycin are not good options, but a prolonged course of linezolid could be the best therapy [19].

### 2.2. Eyes Infections

Previously, the implication of CoNS in the pathogenesis of a corneal ulcer was ignored because these organisms are part of normal flora and ubiquitous. However, improvements in diagnosis and identification have revealed that CoNS are an important cause of infected corneal ulcers. Among CoNS, *S. haemolyticus* is the second most prevalent species causing eye infections [25,26]. Makki et al. reported that 36% of the ocular infections were caused by *S. haemolyticus* [27]. Likewise, Wong et al. reported that *S. haemolyticus* causes endophthalmitis mainly post-operation [28]. In the previous study, the authors described a case of endophthalmitis following femtosecond cataract surgery caused by *S. haemolyticus* that was associated with a progressive infection and severe inflammation on the first day following the operation [28]. Interestingly, *S. haemolyticus* isolates of multilocus sequence type 25 (ST25) which belong to clonal complex 1 (CC1) are reported as a causative agent of keratitis in India and Europe [29,30]. Moreover, *S. haemolyticus*, together with *S. epidermidis*, were also isolated from intraocular lenses [31,32]. *S. haemolyticus* isolates that cause ocular infection have intercellular adhesion (ica) properties due to the formation of biofilm which is composed of the extracellular DNA (eDNA) and protein [33]. The presence of biofilm and expression of quorum sensing are the main features of *S. haemolyticus* isolates causing ocular infections [33].

### 2.3. Nosocomial Infection

CoNS are common skin commensals that start to colonize the body surfaces very early in life. After 48 h of birth, about 100% of infants acquire CoNS during passage through the birth canal or by contacting nursery personnel [34]. The most common colonizing species are *S. epidermidis*, *S.warneri*, and *S. haemolyticus* [35]. *S*. *haemolyticus*, together with *S*. *epidermidis* and *S*. *hominis,* were the prevalent staphylococci species detected in surfaces that are touched at a high frequency in the community and hospitals in London [36]. Similarly, *S. haemolyticus* and *S. epidermidis* were the most common CoNS isolates (34% and 27%, respectively) detected in different hospital wards in Iran [37]. Genotyping studies showed that *S. haemolyticus*, also *S. epidermidis*, colonizing the GIT of newborns are responsible for late-onset sepsis in the preterm neonates [38]. Moreover, Perdreau-Remington et al. reported that a single clone of *S. haemolyticus* showed widespread dissemination among the hands of medical personnel and in different places and wards [39]. Collectively, the previous findings suggest the dissemination of *S. haemolyticus* in hospitals which explains the nosocomial infection associated with *S. haemolyticus.*

*S. haemolyticus* is an important causative pathogen of hospital-acquired infections, particularly in a neonatal ICU [40]. The presence of venous catheters or medical devices increases the risk of infections [40,41]. In addition, an outbreak caused by *S. haemolyticus* was recorded in an Italian intensive care unit [42]. The nosocomial isolates of *S. haemolyticus* showed the highest level of resistance to antibiotics among most members of the CoNS [43]. During hospitalization, an increase in the rate of antibiotic resistance is observed, particularly in the methicillin-resistant strains of *S. haemolyticus* [39]. Moreover, vancomycin and teicoplanin-resistant *S. haemolyticus* strain was recorded in a patient with myelogenous leukemia who suffered from a septic episode during the cytostatic course [44]. Furthermore, *S. haemolyticus* isolates that caused an outbreak in Italy were resistant to linezolid [42]. These multidrug-resistant skin colonizing bacteria are not only at risk for the emergence and spread of nosocomial infections but can also infect healthcare personnel and patient visitors [45].

Furthermore, *S. haemolyticus* can resist disinfection. Molecular typing of infections caused by MRSH collected over a 3 year period from the neonatal ICU revealed that *S. haemolyticus* can survive in disinfectant solutions which consequently can act as a reservoir for infecting the newborns, pointing to the importance of testing the ability of disinfectants in neonatal ICUs against these pathogens [9].

The factors that affect the survival and spread of multi-drug resistant *S. haemolyticus* isolates in hospitals are not completely known. Bouchami and colleagues reported that the insertion sequence transposition (mainly IS1272) and chromosomal rearrangement and recombination processes in *S. haemolyticus* is one strategy that helps in the bacterial evolution, adaptation, pathogenesis, and survival in the hospitals, hence causing nosocomial infections [46].

### 2.4. Male Infertility

*S. haemolyticus* causes infection of the male genital system and it could be responsible for male infertility. *S. haemolyticus* infection decreases sperm motility and viability [47,48]. In addition, contact of *S. haemolyticus* with the ejaculated spermatozoa can affect the architecture of the sperm plasma membrane and therefore lead to male infertility [47,48]. Moreover, exposure of human spermatozoa to *S. haemolyticus* leads to an increase in phosphatidylserine externalization, DNA fragmentation, and the percentage of apoptotic as well as necrotic sperm cells [49]. Furthermore, *S. haemolyticus* infection decreases the percentage of sperm with normal mitochondrial transmembrane potential [49]. Pindar and Viau described a case of *S. haemolyticus* bacteremia secondary to epididymo-orchitis, and there is no involvement of venous or urinary catheters [50].

### 2.5. Other Human Diseases

In addition to the aforementioned clinical conditions, *S. haemolyticus* infection is also recorded in different human infections such as chronic prostatitis [51], coeliac disease [52], community-acquired skin, and soft-tissue infections [53], and continuous ambulatory peritoneal dialysis-associated peritonitis [22]. In addition, *S. haemolyticus* are among the predominant organisms colonizing the periurethral and urethra in males and females, they regularly account for about 10% of UTIs [54,55]. Furthermore, *S. haemolyticus*-associated ventricular atrial shunt nephritis was recorded [56]. Moreover, *S. haemolyticus* causes meningitis in an allogeneic stem cell transplant patient following central catheter-related bacteremia with no previous history of neurosurgical procedures [19]. Furthermore, *S. haemolyticus* was isolated from a 73-year-old man who presented with liver abscess and silent colon cancer [57].

### 2.6. Animal Disease

Methicillin-resistant *S. haemolyticus* (MRSH) was isolated from dogs, but not from cats or horses [14]. MRSH was also isolated from pure-breed kennels and a kennel owner [14]. The previous findings suggest the possibility of bacteria transmission from the animals to the owners and the veterinary personnel. Importantly, the isolates that infect humans and animals are highly resistant to available antibiotics including β-lactams, macrolides, gentamicin, and tetracycline [14]. Moreover, multidrug-resistant *S. haemolyticus* was found in animal food suggesting that these bacteria could aid in the spread of resistance to antimicrobial agents in the places of food manufacture [58].

## 3. Antibiotic Resistance in *S. heamolyticus*

Over recent years, different investigators have described an increasing frequency of multidrug-resistant strains of *S. haemolyticus* [16,59,60]. *S. haemolyticus* is notably more resistant to antibiotics than any other CoNS, and the widest spectrum of resistance was observed among strains isolated from the hospital environment [60,61,62]. The presence of resistance genes in *S. haemolyticus* and its spread in the hospital environment constitutes a potential risk since this bacterium can store the resistance genes and transmit them to other species [63]. Bakthavatchalam et al. used next-generation sequencing technology to characterize the whole genome of multidrug-resistant *S. haemolyticus*, and they characterized three antibiotic-resistant genes. The first two genes named *blaZ* and *norA* are responsible for resistance to β-lactam and quinolone, respectively. The third gene “*msr (A)*” mediates the cross-resistance to different antimicrobial agents such as macrolides, lincosamide, and streptogramin B [64]. The genome of *S. haemolyticus* contains large quantities of the mobile genetic elements (insertion sequences, IS) such as IS256 and IS1272 which participate in the bacterial evolution, shaping the population structure through the DNA recombination process [46]. Moreover, these IS play an important role in bacterial adaption to host and hospital environment and genome flexibility [46]. Furthermore, they could transfer the drug resistance to other staphylococcal species, as shown that the sequences of beta-lactamase and *qacA* genes were identical in both *S. aureus* and *S. haemolyticus* indicating interspecies transfer of IS between these species [64,65,66,67]. Similarly, Kim and Jang recognized the integration of *S. aureus* plasmid, pS0385-1 into the chromosome of *S. haemolyticus* IPK_TSA25 and this integration confers the resistance to antibiotics mainly tetracyclines [68].

Furthermore, the efflux pump mechanism is documented in *S. haemolyticus* human clinical isolates, and it is associated with resistance to gentamicin, erythromycin, ciprofloxacin, chloramphenicol, and tetracycline [69]. The multidrug-resistant pump is mediated by several genes such as *qacG*, *qacH*, and *qacJ* genes [69]. Interestingly, the *qac* genes also confer the resistance of *S. haemolyticus* to antiseptics, and these resistance genes can be horizontally transferred among bacteria [69]. Table 1 summarizes the resistance of *S. haemolyticus* to common antibiotics. 

### 3.1. β-Lactam

Some isolates of *S. haemolyticus* are not susceptible to β-lactams. Barros and colleagues studied the antibiotic profile of 64 clinical isolates of *S. haemolyticus.* They found that 95% of the isolates were resistant to penicillin and ampicillin and 88% of the isolates were resistant to oxacillin and cefoxitin [80]. Similarly, Manoharan et al. recorded that the susceptibility of 356 clinical isolates of *S. haemolyticus* to cefoxitin and penicillin was very low, 8.7% and 5.9%, respectively [81]. Likewise, De Vecchi et al. showed that *S. haemolyticus,* isolated from joint infections, have the highest level of oxacillin resistance (83%), compared to *S. aures* and other CoNS species isolated from the same patients [82]. MRSH became the reason for a significant limitation in the use of β-lactam antibiotics [62].

### 3.2. Methicillin

Methicillin-resistant CoNS was first isolated in 1961 in a clinical laboratory in the UK at rates higher than in *S. aureus* [83]. Nevertheless, CoNS was not thought to be particularly pathogenic to human beings at that time.

Both *S. aureus* and CoNS harbor the *mecA* gene. Therefore, they share the resistance mechanism to methicillin which is mediated by modified transpeptidase enzymes (PBP2a) that crosslink the peptidoglycan layers by synthesizing the pentaglycine bridges in peptidoglycan. This modified PBP2 binds the methicillin at a much lower affinity leading to therapeutic failure [70].

The sequences of the *mecA* gene of *S. aureus*, *S. haemolyticus,* and *S. epidermidis* are similar by 99.95%. Such a degree of similarity supports the hypothesis of the interspecies transfer of the *mecA* gene. Unfortunately, the investigated CoNS groups are often not specified to the species levels, which hinders the accurate evaluation of *S. haemolyticus* resistance rates to methicillin [84]. Molecular analysis demonstrated that the *S. haemolyticus* genome comprises the *ccr* gene complex that contains a chromosomal recombinase, which enables the combination of the mec cassette with chromosomal DNA, and sometimes also other resistance and virulence genes [85,86]. Both the *mec* gene and the *ccr* gene complex form the staphylococcal cassette chromosome mec (SCCmec) cassettes that mediate bacteria pathogenesis [87]. In *S. aureus*, about 11 types of SCCmec cassettes have been identified. The largest diversity of SCCmec sequences is observed amongst *S. epidermidis, S. haemolyticus,* and *S. hominis* strains [55]. SCCmec types III, IV, and V were detected in methicillin-resistant CoNS and some bacteria contain several types [87]. In *S. haemolyticus*, type V is the most frequently identified SCCmec cassette [55,87]. Importantly, MRSH isolates are widespread in the hospital environment. In South Korea, 51.4% of X-ray cassettes were contaminated with genetic similarities to MRSH strains [88]. In another study, 96% of *S. haemolyticus* strains were resistant to methicillin among Brazilian isolates [12]. MRSH strains were detected in 67.5% of CoNS isolated from patients with nosocomial bacteremia in ICU in Istanbul [89].

The ability of *S. haemolyticus* to transfer genes to other species was elucidated at the neonatal ICU in Orebro University Hospital in Sweden in 2008 where a case of SCCmec type V cassette was transferred from MRSH to methicillin-susceptible *S. aureus* [90]. During 20 years of analysis in Zurich, Switzerland between 1986 and 2005, the detection rate of methicillin resistance CoNS bacteria jumped five folds [91].

Some recent studies showed that the incidence of oxacillin resistance of *S. haemolyticus* isolates has exceeded 80% [92,93]. Some strains of *S. haemolyticus* were even resistant to ceftobiprole which is a fifth-generation cephalosporin. These strains had MIC ranges of 1 to 4 µg/mL [94].

### 3.3. Glycopeptides

Glycopeptides are given to patients with severe infections caused by multidrug-resistant CoNS. Glycopeptide antibiotics block the late stages of peptidoglycan cross-linking by binding to the dipeptide terminus D-Ala-D-Ala leading to the inhibition of bacterial cell wall synthesis [95]. Vancomycin and teicoplanin are members of available glycopeptides. The bacterial resistance to glycopeptides was developed due to the uncontrolled use [96,97]. Glycopeptide heteroresistance is common in MRSH, but rare in methicillin-susceptible *S. haemolyticus* [64]. Bakthavatchalam et al. reported the first isolate of methicillin-susceptible *S. haemolyticus* was in India which is resistant to teicoplanin and with decreased sensitivity to vancomycin [64]. The resistance of *S. haemolyticus* to glycopeptide in the previous case is due to the alteration of the glycopeptide resistance-associated histidine kinase (GraS) mediated by the insertion of two amino acids (leucine and proline) at adjacent sites inside the GraS target. Furthermore, Billot-Klein et al. showed that the alteration in the cross-bridge between the peptidoglycan layers of *S. haemolyticus* decreases the efficiency of binding of glycopeptides to the bacterial cell wall leading to antibiotic resistance [71].

#### 3.3.1. Vancomycin

In 2002, in the US, the first vancomycin-resistant *S. aureus* was reported which contained the *vanA* gene. Up to the present, the frequency of staphylococci resistance to vancomycin is infrequently reported [98]. The resistance of *S. haemolyticus* to vancomycin was recorded earlier. Vancomycin-resistant *S. haemolyticus* isolates were first recorded in a patient with peritonitis, these isolates showed resistance to vancomycin in vivo and in vitro [99]. The exact mechanism of CoNS resistance to vancomycin remains unclearly defined. One of the reported mechanisms for the reduced susceptibility of *S*. *epidermidis* and *S. haemolyticus* to vancomycin is the increase in cell wall thickening [100,101]. Some vancomycin-resistant CoNS had an overproduction of cell wall peptidoglycan material leading to an excess of glycopeptide binding sites [102]. Therefore, it seems that *S. aureus* and CoNS share the same mechanism of reduced susceptibility to glycopeptides.

One of the disadvantages of vancomycin is its reduced activity on biofilms and its low intracellular penetration power. In contrast, rifampicin is very effective in the eradication of biofilms caused by *Staphylococci*. However, it should be given with other antibiotics to avoid the rapid development of resistance [11].

#### 3.3.2. Teicoplanin

Teicoplanin is effective in the treatment of *S. haemolyticus* infections, with rare homogenous resistance reports [103]. However, compared with other CoNS, teicoplanin is less potent in vitro against *S. haemolyticus* isolates [21]. The first cases of teicoplanin-resistant *S. haemolyticus* were described in the US and UK in 1986. *S. haemolyticus* isolated from both reports were also resistant to methicillin but sensitive to vancomycin [103,104]. *S. haemolyticus* which developed resistance to teicoplanin was also resistant to vancomycin [104]. Several reports showed that the rate of resistance to teicoplanin is higher than vancomycin among *S. haemolyticus* isolates [21,105,106].

During the period 2000–2003, *S. haemolyticus* was the second most frequent organism of CoNS isolated from patients with bacteremia (following *S. epidermidis*). Teicoplanin resistance was detected in 11–29% of these isolates [66]. Bakthavatchalam and colleagues reported that teicoplanin resistance operon (tcaRAB) plays a crucial role in the resistance of *S. haemolyticus* to teicoplanin [64]. Substitutions in three amino acids in tcaA (I3N, I390N, and L450I), and/or mutations in the transcriptional regulator tcaR (L44V, G52V, and S87P) were associated with the resistance of *S. haemolyticus* to teicoplanin [64].

### 3.4. Linezolid

Linezolid is recommended in cases of severe bacterial infections caused by methicillin-resistant staphylococci or vancomycin-resistant bacteria [61,72]. Linezolid inhibits bacterial protein synthesis by interfering with the peptidyl transferase of 23S rRNA in the 50S ribosomal subunit [107]. Linezolid-resistant *S. haemolyticus* has been isolated from four patients’ pus samples. Two samples were isolated from patients with chronic osteomyelitis, and two isolates were detected in cases of pemphigus vulgaris [74]. Some *S. haemolyticus* variants show a mucoid appearance. These mucoid colonies were associated with linezolid-resistant and were difficult to treat [108]. Linezolid-resistant *S. haemolyticus* isolates were responsible for an outbreak in an Italian intensive care unit [42]. The resistant isolates harbored the G2576T mutation that confers the resistance to linezolid which was retained for several passages [42]. *S. haemolyticus* isolates that carry the G2576T mutation in the *23S rRNA* gene can disseminate in the hospital and ICU and are associated with the spread of nosocomial infections [61,109]. Furthermore, Kumari and colleagues described several mutations such as G2576T, G2447U, and C2534U at the domain V of the *23S ribosomal RNA* gene and are associated with linezolid resistance [73]. Interestingly, the previous mutations were recorded in multiple, not single, clones of *S. haemolyticus* and were not associated with inappropriate use of linezolid [73]. Importantly, all linezolid-resistant *S. haemolyticus* isolates carry the *cfr* gene which confers methylation of 23S ribosomal RNA at A2503 and exhibits resistance to chloramphenicol, florfenicol, clindamycin, streptogramin A, and linezolid [74,75,110]. The previous findings suggest that the use of linezolid especially in the case of *S. haemolyticus* should be controlled to preserve the drug clinical utility.

### 3.5. Lincosamides

Lincosamides such as lincomycin, clindamycin, and Pirlimycin are effective against gram-positive cocci, and they suppress the bacterial protein expression by acting on the 50s ribosome. *S. haemolyticus* was resistant to high levels of lincomycin through plasmid-mediated inactivation of the lincomycin and clindamycin [111]. The enzyme responsible for the inactivation of lincosamides is the lincosamide O-nucleotidyltransferase that is encoded by *lnu* (*A*) and *lnu* (*A′*) (formerly *lin*) genes [76,77]. Novotna et al. identified *S. haemolyticus* isolates that are resistant to both clindamycin and lincomycin but sensitive to erythromycin [112]. The mechanism of resistance to lincosamides in the previous isolates was not identified, and it was not related to the resistance gene, ribosomal mutation, and/or inactivation resistance [78,112]. Further study showed that the resistance of these isolates to lincosamides was due to efflux of the drug which was mediated by the *vga (A)_LC_* resistance gene, that formed the ATP-binding cassette (ABC) family [78]. The mechanism of resistance of *S. haemolyticus* to lincosamides via efflux mechanism is similar to the resistance of Staphylococci to macrolide-streptogramin B via by *Msr (A)* gene [78].

### 3.6. Mupirocin

Mupirocin is an intranasal antibiotic used for the eradication of staphylococci infection. Mupirocin targets isoleucyl-tRNA synthetase which is required for protein synthesis, and resistance to this antibiotic arise from mutation in isoleucyl-tRNA (low level of resistance) or similarity between this target and eukaryotic enzymes (high level of resistance) [79]. The resistance to mupirocin is recorded in *S. haemolyticus* isolates due to the presence of the *mupA* gene in mupirocin resistance (Mup^R^) plasmids [13]. Interestingly, an insertion sequence (IS257) is flanking the *mupA* gene which could aid in the horizontal transfer of *S. haemolyticus mupA* gene to the environment or other bacteria [13]. Rossi and colleagues reported the transfer of Mup^R^ from *S. haemolyticus* clinical isolates to *S. aureus* suggesting that *S. haemolyticus* act as a reservoir for Mup^R^ [67]. Not only in humans but high levels of Mup^R^ were recorded in MRSH in healthy and diseased dogs [113,114].

## 4. Virulence Factors of *S. haemolyticus*

**Figure 2:**
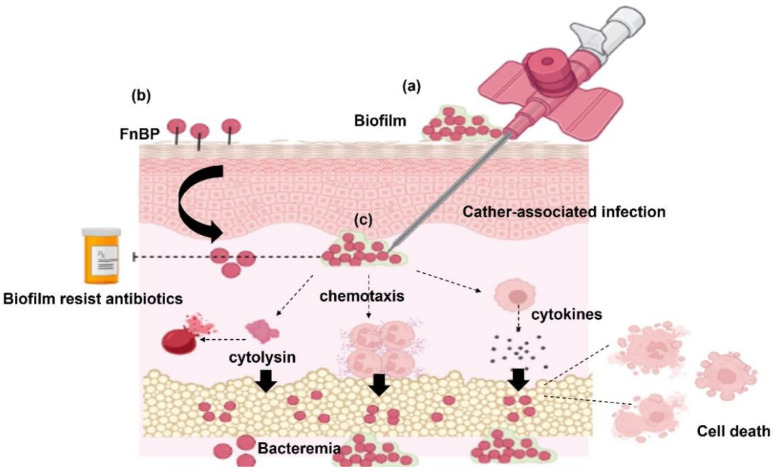
Pathogenesis of *S. haemolyticus.* (**a**) *S. haemolyticus* isolates that form a biofilm adhere to the catheter and internalize with it inside the host. Biofilm-associated *S. haemolyticus* isolates are resistant to antibiotics. (**b**) Fibronectin-binding proteins (FnBP) of *S. haemolyticus* help in bacterial adherence, internalization, and invasion to host cells. (**c**) *S. haemolyticus* invades the host cells causing bacteremia through the release of cytolysins, proinflammatory cytokines from the host immune cells, and activation of chemotaxis.

Genome sequencing of common *S. haemolyticus* strain C10A has illustrated the brief detection of multiple antibiotic resistance and virulence genes [115]. However, many of these virulence factors remained virtually unexplored [116]. Surface substances and cytolysins have crucial effects on the virulence of *S. haemolyticus* [62]. Bacterial adherence and internalization are mediated by biofilm and fibronectin-binding proteins (FnBP). Following the entry to the host cell, toxins and enzymes are released by *S. haemolyticus* which mediate tissue damage, activation of proinflammatory cytokines, and apoptosis of host cells. Figure 2 summarizes the virulence factors that are associated with the pathogenesis of *S. haemolyticus*.

### 4.1. Biofilm Formation

Biofilm is a polysaccharide layer produced extracellularly and aids bacterial attachment to surfaces and medical devices. *S. haemolyticus* isolates that form a biofilm participate in catheters and other devices associated with infections [117,118,119,120]. Biofilm-producing *S. haemolyticus* causes bacteremia, particularly those associated with the use of catheter-associated infections and nosocomial infections [117,118,119].

The biofilm formation by *S. haemolyticus* is a complex process and is increased in the presence of antimicrobial agents [117]. The impact of antibiotics on the inhibition of biofilm formation is controversial. Pereira-Ribeiro and colleagues reported that the biofilm formation of *S. haemolyticus* on abiotic surfaces was not inhibited by antibiotics such as linezolid, teicoplanin, vancomycin, tigecycline, rifampicin, etc. [117]. While Szczuka and colleagues showed that tigecycline/rifampicin combination (biofilm inhibitory concentration ranged from 0.062 to 1 µg/mL) was more effective than daptomycin/rifampicin combination (biofilm inhibitory concentration ranged from 0.125 to 2 µg/mL) against *ica*-independent biofilm, produced by *S. haemolyticus* [11]. The formation of biofilm and resistance to antibiotics could be the causes of persistent bacterial infections and survival inside hospitals [121].

The formation of biofilm by *S. haemolyticus* differs from other Staphylococci. The formation of biofilm by *S. epidermidis* and *S. aureus* is mediated by the *ica* operon which codes for the enzymes responsible for the formation of poly-N-acetylglucosamine/polysaccharide intercellular adhesion which participates in the formation of the biofilm matrix [122]. However, the biofilm formation by *S. haemolyticus* is mainly *ica*-independent because no *icaAD* genes were observed in the bacteria [117,118,119]. Moreover, In contrast to *S. aureus* and *S. epidermidis*, *S. haemolyticus* biofilms do not include accumulation-associated protein and biofilm-associated protein genes and are independent of polysaccharide intercellular adhesion (PIA) [118].

Still the process of biofilm formation by *S. haemolyticus* has not yet been extensively studied. Further studies need to verify the detailed steps of biofilm formation in *S. haemolyticus* and the factors regulating it.

### 4.2. S. haemolyticus Surface Proteins Required for Bacteria Adherence

Besides the biofilm formation, *S. haemolyticus* secretes fibronectin-binding proteins (FnBP) that play an important role in bacterial adherence to the extracellular matrix, bacterial internalization into the host cell, and invasion [15]. The adherence property of clinical *S. haemolyticus* is different from commensal *S. haemolyticus.* Commensal *S. haemolyticus* have high adherence to fibronectin and collagen, while clinical *S. haemolyticus* have low adherence to fibronectin and collagen [123]. However, using the bacteria surface shaving approach, 65 surface proteins were identified in clinical *S. haemolyticus* isolates and were associated with adherence to human keratinocytes such as the bacterial Toll/interleukin-1 like (TIRs) domain-containing protein, the bifunctional autolysin Atl, LPXTG, and the transglycosylase SceD [123].

### 4.3. Toxins and Enzymes

Some *S. haemolyticus* isolates secrete enterotoxins and/or hemolysins [55,124,125]. Staphylococci enterotoxins act as superantigens that activate the immune cells to produce their cytokines resulting in food poisoning and other diseases such as sepsis and multiorgan dysfunction [126,127]. Several enterotoxin genes were recorded in *S. haemolyticus* such as *sea*, *seb*, *sec*, *seg*, and *sei* and one or more genes could be recorded in the isolates found from blood cultures [125]. *S. haemolyticus* isolates containing enterotoxin genes are associated with bovine mastitis [128] and peritonitis in continuous ambulatory peritoneal dialysis (CAPD) patients [129]. In addition, enterotoxin-producing *S. haemolyticus* was isolated from the clinical samples of newborns [130]. Cytotoxins (also known hemolysins) are virulence factors associated with the pathogenesis of *S. aureus*, but data about the effect of these toxins in CoNS infections is unknown. There are several hemolysins associated with Staphylococci infections including α-hemolysin, β-hemolysin, and δ-hemolysin. α-hemolysin producing *S. aureus* strains that express a high level of *hla* gene, causes damage to the skin, neurons, epithelium, endothelial and immune cells, while the strains that are deficient in *hla* gene are less virulent [131,132]. Interestingly, more than 90% of *S. haemolyticus* harbor *hla* gene [125], and these isolates are associated with diabetic ulcers [133]. In addition, β-hemolysin and δ-hemolysin were detected in 81% and 40.5% of the *S. haemolyticus* isolates, and both toxins were recorded in 30% of the isolates [125]. β-toxin plays a role in the evasion of the pathogen to the immune system and scavenging of nutrients [134], while δ-toxin is encoded by regulatory RNAIII and affects the agr quorum-sensing system [135]. Moreover, Da and colleagues identified phenol-soluble modulins (PSMs) in *S. haemolyticus* isolates, and these toxins have a broad cytolytic activity [116]. Alpha-type PSM (PSMα) has a potent leucocidin and hemolytic activity, and β-type PSM has anti-gonococcal activity [116]. Furthermore, *S. haemolyticus* PSMs induce neutrophil chemotaxis resulting in a pronounced pro-inflammatory effect [116]. The previous findings highlight the importance of toxins in the invasion of *S. haemolyticus* and the development of bacteremia and sepsis.

### 4.4. Cytotoxicity and Apoptosis of the Host Cells

In vitro studies showed that *S. haemolyticus* infection could alter the host immune response through its effect on the host cells [15,136]. Krzymińska et al. showed that *S. haemolyticus* causes injury and loss of mitochondrial membrane potential in macrophages [136]. In addition, *S. haemolyticus* infection is cytotoxic to the macrophages through induction of caspase-dependent apoptosis [136]. The previous findings show one strategy for *S. haemolyticus* persistence and dissemination in the host through inhibition and host macrophages. Similarly, Eltwisy et al. showed that *S. haemolyticus* infection causes damage and apoptosis of primary human skin fibroblast cells, and it induces the release of proinflammatory cytokines from the PBMCs cocultured with the skin fibroblast [15]. Collectively, the previous reports show that *S. haemolyticus* infection causes damage to the host cells through apoptosis.

## 5. Conclusions and Future Perspectives

Human skin is colonized by an opportunistic bacterial pathogen *S. haemolyticus* which carries antibiotic resistance genes. *S. haemolyticus*, especially the clinical isolates, are mainly multidrug-resistant, and these isolates produce biofilms, toxins, and enzymes leading to infections that are difficult to treat. The increasingly growing spread of multidrug-resistant *S. haemolyticus* in the hospital environment could have potentially devastating complications. The presence of resistance genes in *S. haemolyticus* (Table 1) suggests the possibility of resistance gene transfer between *S. haemolyticus* and other bacteria which explains the widespread resistance to antibiotics and the survival in the hospitals. Still, not all the mechanisms of *S. haemolyticus* resistance to antibiotics are known, and future studies need to verify other resistance mechanisms. Importantly, the uncontrolled use of antibiotics aids in the spread of resistant *S. haemolyticus* isolates. Therefore, the use of antibiotics, especially in the case of *S. haemolyticus,* should be controlled to preserve the drug’s clinical utility.

## Figures and Tables

**Table 1:** Resistance of *S. haemolyticus* to antibiotics.

Antibiotic	Antibiotic Action	Resistance Gene	Mechanism of Resistance	Reference
**Methicillin**	Inhibits bacterial peptidoglycan cross-linking through inhibition of transpeptidase enzyme	*MecA*	Modification of the transpeptidase enzyme that causes lower affinity to the drug.	[70]
**Glycopeptides** **(Vancomycin and teicoplanin)**	Binds to the D-Ala-D-Ala leading to suppression of bacterial cell wall synthesis	*GraS*	Alteration of GraS target by insertion of leucine and proline aa at positions 315 and 316, respectively.	[64,71]
		*TcaRAB* including *tca* and *tcaR*	Substitutions in 3 amino acids in tcaA (I3N, I390N, and L450I). mutations in the tcaR (L44V, G52V, and S87P).	
**Linezolid**	Inhibits bacterial protein expression via interfering with the 23S rRNA in the ribosome	Domain V region of *23S rRNA* gene. *cfr* gene.	Modification of the ribosomal peptidyl transferase center region due to several mutations in 23S rRNA region such as G2576T, G2447U, and C2534U mutations. Mutations in ribosomes such as L3. Methylation at the ribosomal site.	[42,72,73,74,75]
**Lincosamides** (lincomycin, clindamycin, Pirlimycin)	Interferes with bacterial protein formation through binding to the 23S/ 50s ribosome	*lnu(A)* and *lnu(A′)vga(A)_LC_*	Inactivation of lincosamides via lincosamide O-nucleotidyltransferase enzyme. Efflux of lincosamides through ABC family.	[76,77]
				[78]
**Mupirocin**	Inhibits bacterial protein synthesis via targeting isoleucyl-tRNA synthetase	*mupA*	Mutation in isoleucyl-tRNA Similarity between bacterial isoleucyl-tRNA and eukaryotic enzymes	[79]

## Data Availability

Not applicable.

## Abbreviations

Aap	accumulation-associated protein
Ala	alanine amino acid
Bhp	biofilm-associated protein
BSIs	Bloodstream infections
CC1	clonal complex 1
CAPD	continuous ambulatory peritoneal dialysis
CoNS	Coagulase-negative Staph
CRS	catheter-related sepsis
eDNA	extracellular DNA
FnBP	fibronectin-binding proteins
GraS	glycopeptide resistance-associated histidine kinase
ica	intercellular adhesion
ICU	intensive care unit; IS: insertion sequence
MIC	minimum inhibitory concentration
MRSH	methicillin-resistant S. haemolyticus
PBP	penicillin-binding protein
PIA	polysaccharide intercellular adhesion
PSMs	phenol-soluble modulins
tcaRAB	teicoplanin resistance operon
tcaR	transcriptional regulator
TIRs	Toll/interleukin-1 like
SCCmec	Staphylococcal cassette chromosome mec
ST25	sequence type 25
S.	Staphylococcus.
UTIs	urinary tract infections
